# Abemaciclib抑制c-Myc高表达小细胞肺癌增殖、侵袭和迁移的生物学功能的研究

**DOI:** 10.3779/j.issn.1009-3419.2023.106.04

**Published:** 2023-02-20

**Authors:** Jingjing GUO, Di MU, Wenwen YU, Leina SUN, Jiali ZHANG, Xiubao REN, Ying HAN

**Affiliations:** ^1^300060 天津，天津医科大学肿瘤医院生物技术研究室（郭晶晶，牟迪，于文文，任秀宝，韩颖），病理科（孙蕾娜），生物治疗科（张家丽，任秀宝，韩颖）; ^1^Department of Immunology,; ^2^国家恶性肿瘤临床医学研究中心; ^2^Department of Pathology; ^3^天津市肿瘤防治重点实验室,天津市恶性肿瘤临床医学研究中心,天津市肿瘤免疫与生物治疗重点实验室; ^3^Department of Biotherapy, Tianjin Medical University Cancer Institute and Hospital, National Clinical Research Center for Cancer, Tianjin Key Laboratory of Cancer Prevention and Therapy, Tianjin’s Clinical Research Center for Cancer, Tianjin Key Laboratory of Cancer Immunology and Biotherapy, Tianjin 300060, China

**Keywords:** 肺肿瘤, 细胞周期依赖性激酶4/6, c-Myc, Lung neoplasms, Cyclin-dependent kinases 4 and 6, c-Myc

## Abstract

**背景与目的:**

c-Myc高表达小细胞肺癌（small cell lung cancer, SCLC）因易复发转移导致患者生存率极低，细胞周期依赖性激酶4和6（cyclin-dependent kinases 4 and 6, CDK4/6）抑制剂Abemaciclib在多种肿瘤的治疗中均起到关键作用，但其对SCLC的作用及机制仍不明晰。本研究旨在分析Abemaciclib抑制c-Myc高表达的SCLC增殖、侵袭和迁移的作用和分子机制，以期为减少患者的复发和转移拓展新的方向。

**方法:**

通过STRING数据库分析了CDK4/6和c-Myc蛋白相互作用网络，免疫组化实验分析了31例SCLC癌组织和配对的相邻正常组织中CDK4/6及c-Myc的表达，CCK-8实验和平板克隆实验检测Abemaciclib对SCLC增殖的影响，Transwell及划痕实验检测Abemaciclib对SCLC的侵袭和迁移能力的影响，Western blot检测Abemaciclib对CDK4/6及相关转录因子的影响，流式细胞术分析了Abemaciclib对SCLC的细胞周期和免疫检查点的调节作用。

**结果:**

通过STRING蛋白相互作用网络分析发现CDK4/6的表达均与c-Myc相关，c-Myc可以直接调节ASCL1（achaete-scute complex homolog 1）、NEUROD1（neuronal differentiation 1）和Yes相关蛋白1（Yes-associated protein 1, YAP1）的表达，CDK4和c-Myc均调控程序性死亡配体1（programmed cell death ligand 1, PD-L1）的表达。通过免疫组化实验发现，CDK4/6及c-Myc在SCLC癌组织中的表达高于癌旁组织（P<0.0001）。CCK-8实验、平板克隆实验、Transwell实验及划痕实验验证了Abemaciclib可以有效地抑制SBC-2和H446^OE^的增殖、侵袭和迁移（P<0.0001）。Western blot进一步分析了Abemaciclib在抑制SBC-2和H446^OE^细胞内CDK4（P<0.05）和CDK6（P<0.05）的同时，也影响了c-Myc（P<0.05）、ASCL1（P<0.05）、NEUROD1（P<0.05）和YAP1（P<0.05）等与SCLC浸润和转移相关的蛋白。流式细胞术发现Abemaciclib不仅抑制SCLC的细胞周期进程（P<0.0001），还可以提高SBC-2（P<0.01）和H446^OE^（P<0.001）细胞表面PD-L1的表达。

**结论:**

Abemaciclib明显抑制SCLC的增殖、侵袭、迁移和细胞周期进程，其通过抑制CDK4/6下调c-Myc、ASCL1、YAP1和NEUROD1蛋白表达的同时，也提高了PD-L1的表达。

## Abstract (en)

**Background and objective:**

Small cell lung cancer (SCLC) with high c-Myc expression is prone to relapse and metastasis, leading to extremely low survival rate. Cyclin-dependent kinases 4 and 6 (CDK4/6) inhibitor Abemaciclib plays a key role in the treatment of tumors, but the effects and mechanisms on SCLC remain unclear. This study was to analyze the effect and molecular mechanism of Abemaciclib in inhibiting proliferation, migration and invasion of SCLC with high c-Myc expression, with a view to expanding a new direction for reducing the recurrence and metastasis.

**Methods:**

Proteins interacting with CDK4/6 were predicted using the STRING database. The expressions of CDK4/6 and c-Myc in 31 cases of SCLC cancer tissues and paired adjacent normal tissues were analyzed by immunohistochemistry. The effects of Abemaciclib on the proliferation, invasion and migration of SCLC were detected by CCK-8, colony formation assay, Transwell and migration assay. Western blot was used to detect the expressions of CDK4/6 and related transcription factors. Flow cytometry was used to analyze the effects of Abemaciclib on the cell cycle and checkpoint of SCLC.

**Results:**

The expression of CDK4/6 was associated with c-Myc by STRING protein interaction network. c-Myc can directly modalize achaete-scute complex homolog 1 (ASCL1), neuronal differentiation 1 (NEUROD1) and Yes-associated protein 1 (YAP1). Moreover, CDK4 and c-Myc regulate the expression of programmed cell death ligand 1 (PD-L1). Immunohistochemistry showed that the expressions of CDK4/6 and c-Myc in cancer tissues were higher than those in adjacent tissues(P<0.0001). CCK-8, colony formation assay, Transwell and migration assay verified that Abemaciclib could effectively inhibit the proliferation, invasion and migration of SBC-2 and H446^OE^(P<0.0001). Western blot analysis further showed that Abemaciclib not only inhibited CDK4 (P<0.05) and CDK6 (P<0.05), but also affected c-Myc (P<0.05), ASCL1 (P<0.05), NEUROD1 (P<0.05) and YAP1 (P<0.05), which are related to SCLC invasion and metastasis. Flow cytometry showed that Abemaciclib not only inhibited the cell cycle progression of SCLC cells (P<0.0001), but also significantly increased PD-L1 expression on SBC-2 (P<0.01) and H446^OE ^(P<0.001).

**Conclusion:**

Abemaciclib significantly inhibits the proliferation, invasion, migration and cell cycle progression of SCLC by inhibiting the expressions of CDK4/6, c-Myc, ASCL1, YAP1 and NEUROD1. Abemaciclib can also increase the expression of PD-L1 in SCLC.

起源于神经内分泌细胞前体的小细胞肺癌（small cell lung cancer, SCLC）发病率虽然不高，仅次于肺腺癌和肺鳞癌^[1⇓-3]^，但却是最难治的癌症之一。在过去的三十年，以铂类为基础的化疗一直作为SCLC治疗的一线方案。然而，早期复发和转移使得SCLC患者的5年生存率仅有6%^[4,5]^。因此，探究SCLC进展和转移的分子机制，寻求有效地抑制SCLC增殖、侵袭和迁移的分子靶点是目前急需解决的问题。

c-Myc作为MYC家族的重要组成部分，不仅在转录、糖酵解、分化和凋亡等正常细胞增殖过程中起着不可或缺的作用，其过表达还与肿瘤干细胞样细胞和免疫逃逸相关^[6⇓⇓⇓⇓⇓⇓⇓⇓-15]^。研究^[11]^发现，SCLC中最常突变的c-Myc基因影响着ASCL1（achaete-scute complex homolog 1）、NEUROD1（neuronal differentiation 1）和Yes相关蛋白1（Yes-associated protein 1, YAP1）的分化。ASCL1、NEUROD1、POU2F3（POU class 2 homeobox 3）和YAP1作为SCLC差异表达的四种亚型，表现出不同的遗传特征、免疫差异和治疗易感性^[16⇓⇓-19]^，并且不同亚型的SCLC通常以不同比例并存于肿瘤实体中共同促进肿瘤转移。因此，c-Myc或许可以成为SCLC治疗的一个突破点。然而，c-Myc缺乏典型的活性口袋并且没有固定的空间构象导致靶向c-Myc治疗实体肿瘤非常困难^[20]^ 。

Dammert等的研究^[21]^发现c-Myc的过度激活增加了SCLC对细胞周期控制抑制剂的敏感性，揭示了c-Myc在细胞周期进程中的特异调控机制。CDK4/6作为细胞周期进程G_1_期/S期中的重要调节因子，与c-Myc共同促进肿瘤的发生发展，并与患者不良预后相关^[22⇓-24]^。CDK4/6抑制剂Abemaciclib不仅通过下调CDK4/6从而抑制细胞周期进程G_1_期/S期和c-Myc的表达^[25,26]^，还能够抑制CDK4介导的斑点型POZ蛋白（speckle-type POZ protein, SPOP）的磷酸化，从而提高肿瘤细胞表面PD-L1的表达，为SCLC联合应用程序性死亡配体1（programmed cell death ligand 1, PD-L1）抗体提供了一个新的诊疗思路^[27]^。因此，本研究首先探讨了CDK4/6和c-Myc在SCLC恶性发展中的作用，进一步分析了Abemaciclib对c-Myc高表达SCLC增殖、侵袭和迁移的影响及其作用机制。

## 1 材料与方法

### 1.1 STRING数据库分析

通过STRING数据库分析CDK4、CDK6、c-Myc、ASCL1、YAP1、NEUROD1和PD-L1七种蛋白的相互作用，依次输入上述七种蛋白名称，按照物种类型为“Homo sapiens”，置信度为“Medium 0.400”，相互作用为“最大数为10”进行分析。

### 1.2 样本收集

回顾性分析2013年10月-2021年11月天津医科大学肿瘤医院收治的局限期SCLC患者。纳入标准：具有完整的临床资料，包括诊断年龄、性别、吸烟史、转移部位等。排除标准：无明确病理诊断或影像学评估，有急、慢性疾病或其他恶性肿瘤病史者。该研究得到天津医科大学肿瘤医院伦理委员会的批准。

### 1.3 免疫组化染色

免疫组化的一抗包括CDK4（Abcam, 1:200）、CDK6（Abcam, 1:100）和c-Myc（Proteintech, 1:1,000）。石蜡包埋切片在70 °C烘烤2 h-3 h，然后在二甲苯中脱蜡，在乙醇中脱水。切片浸泡在柠檬酸缓冲液或Tris-EDTA中进行微波修复。冷却至室温后，用过氧化氢灭活内源性过氧化物酶，与一抗在4 ^o^C孵育过夜。用磷酸缓冲盐水（phosphate buffered saline, PBS）清洗切片，与未稀释辣根过氧化物酶（horseradish peroxidase, HRP）结合的山羊抗兔抗体二抗体在37 ^o^C孵育30 min后，用3,3'-二氨基联苯啶（diaminobenzidine, DAB）覆盖切片，涂上苏木精，脱水。两位病理医师在不了解标本的临床和病理特征的情况下从每个切片中随机收集至少5张图像，在光镜（Olympus, Japan）下通过染色强度评估组织（评分：阴性为0分，弱为1分，中等为2分，强为3分）和阳性百分比（评分：0%-25%为1分，26%-50%为2分，51%-75%为3分，76%-100%为4分）。

### 1.4 细胞培养和试剂

H446来自美国模式培养物集存库（American Type Culture Collection, ATCC），SBC-2由天津医科大学肿瘤医院黄鼎智教授捐赠，支原体污染均为阴性。H446和SBC-2在RPMI-1640培养基中培养，添加10%胎牛血清和1%青霉素-链霉素。

### 1.5 基因过表达

为了探究Abemaciclib对c-Myc高表达SCLC的影响并验证c-Myc对CDK4/6及其他蛋白的调节作用，我们构建了带有flag标签的c-Myc稳系。c-Myc过表达慢病毒购自中国上海汉恒生物公司。将H446细胞系接种在6孔板上，将2 μg/mL的助转剂和慢病毒加入至不含胎牛血清的1640培养基中。24 h后，用10%胎牛血清的RPMI-1640培养基替换，用2 μg/mL嘌呤霉素筛选感染细胞（H446^OE/NC^）7 d，并维持在1 μg/mL。

### 1.6 CCK-8和平板克隆实验

SBC-2和H446^OE^接种在96孔板上。分别在24 h、48 h、72 h后，用CCK-8孵育2 h后通过分光光度计测量波长为450 nm处的光密度值。Abemaciclib预处理或未处理的细胞接种于6孔板，每孔1,000个细胞，培养2周后用结晶紫染色并采集图像，统计克隆形成率。

### 1.7 细胞侵袭和迁移实验

Transwell实验用来评估细胞侵袭情况。Transwell小室平铺Matrigel 50 μL过夜后，Abemaciclib预处理或未处理的细胞混以无血清培养基加入上室，下室加入含10%胎牛血清的RPMI-1640培养基500 μL。孵育48 h后，用甲醇固定细胞30 min，Giemsa染色10 min-20 min，在100倍镜下拍照。将SBC-2和H446^OE^置于密度为1×10^6^的6孔板中，细胞几乎完全融合。然后用无菌的20 μL移液管尖端制作创面，细胞在低血清或无血清培养基中培养0 h、24 h、48 h拍照，观察细胞迁移情况。

### 1.8 Western blot

用RIPA Lysis Buffer（Beyotime）联合PMSF蛋白酶抑制剂（Thermo Scientific）在100 mm的培养皿中溶解细胞30 min，用Pierce BCA Protein Assay Kit（Thermo Scientific）检测蛋白浓度。这些蛋白样品经电泳、电转移和阻断后与CDK4（Abcam, 1:1,000）、CDK6（Abcam, 1:50,000）、c-Myc（Abcam, 1:1,000）、YAP1（Abcam, 1:5,000）、ASCL1（Abcam, 1:1,000）和NEUROD1（Abcam, 1:1,000）的一抗孵育12 h。TBST洗涤3次后与辣根过氧化物酶标记的二抗室温孵育1 h，TBST再次洗涤3次后用ECL试剂盒进行化学发光显影。应用Image J进行蛋白表达水平分析。

### 1.9 流式细胞术

检测Abemaciclib预处理和未处理的SBC-2和H446^OE^细胞表面的PD-L1（Biolegend）及其细胞周期。所有样品均使用FlowJo软件版本10.6.2（BD）进行分析。

### 1.10 统计学分析

使用软件SPSS 24.0和GraphPad Prism 8，采用t检验分析两组之间的差异。P<0.05被认为具有统计学差异。

## 2 结果

### 2.1 STRING蛋白相互作用网络分析

通过STRING蛋白相互作用网络分析CDK4、CDK6、c-Myc、ASCL1、YAP1、NEUROD1和PD-L1之间的相互作用。如图1所示，MYC与其他几种蛋白相互关联，起着中枢调节作用。CDK4不仅调节CDK6，还与YAP1和PD-L1相互影响。SCLC中两种重要的转录因子ASCL1和NEUROD1也相互调节。

**图1:**
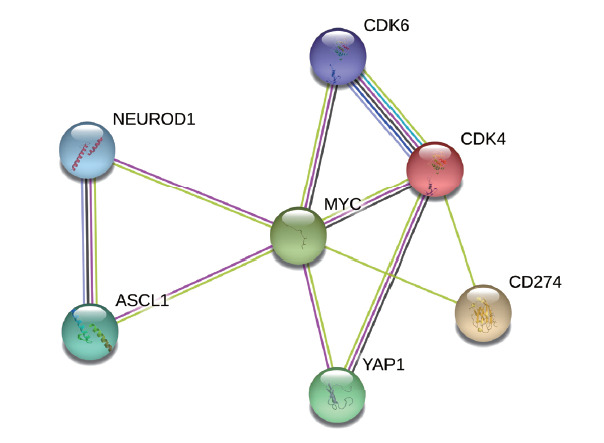
STRING数据库中与CDK4/6基因表达蛋白相关的蛋白网络图

### 2.2 CDK4/6和c-Myc在SCLC中的表达及其与临床预后的关系

基于STRING数据库的分析结果，我们回顾性收集了31例局限期SCLC的组织样本。通过免疫组化染色发现CDK4/6和c-Myc在癌组织中的表达均高于癌旁组织（图2）。

**图2:**
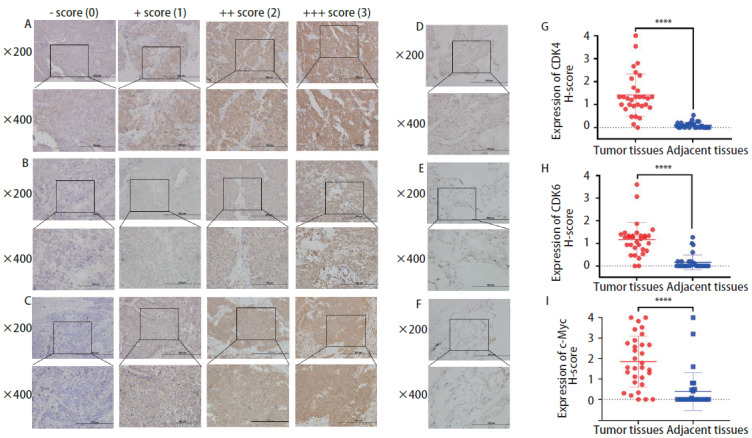
CDK4/6和c-Myc在SCLC组织中的免疫组化染色及评分（×200；×400）。 根据H-score评分标准将CDK4（A）、CDK6（B）和c-Myc（C）在SCLC癌组织染色情况进行评分；D-F分别为CDK4、CDK6和c-Myc在癌旁组织中的染色情况。癌组织中CDK4（G）、CDK6（H）和c-Myc（I）的表达均高于癌旁组织。^****^P<0.0001。

### 2.3 Abemaciclib显著抑制SCLC的增殖、侵袭和迁移

通过CCK-8实验发现Abemaciclib以时间和浓度依赖性的方式抑制SCLC的活性。Abemaciclib对SBC-2的影响在第3天最强（图3A），对H446^OE^的影响更均一（图3B）。另一方面，克隆形成实验（图3C）表明Abemaciclib能够抑制H446^OE^（P<0.0001）和SBC-2（P<0.0001）的增殖。通过分析Transwell实验结果发现Abemaciclib显著抑制了这两种细胞的侵袭（图3D）。如图3E和图3F所示，Abemaciclib抑制了SBC-2和H446^OE^的迁移（P<0.0001）。

**图3:**
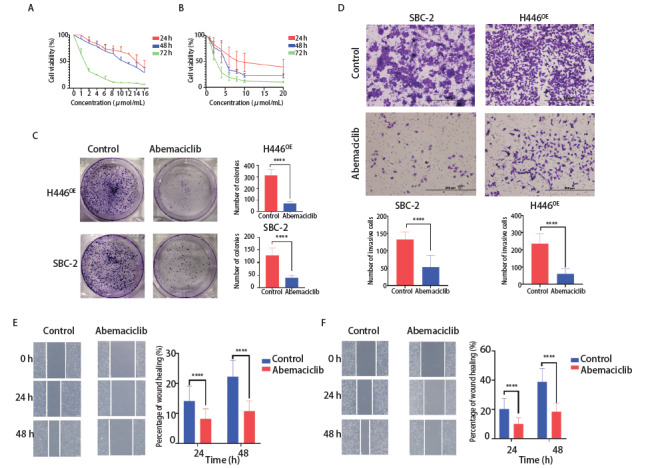
Abemaciclib对SCLC增殖、侵袭和迁移的影响。 A、B：CCK-8实验验证了Abemaciclib对SBC-2（A）和H446^OE^（B）增殖的影响；C：Abemaciclib对SBC-2和H446^OE^细胞集落形成能力的影响；D：SBC-2和H446^OE^未处理或通过Abemaciclib预处理24 h后，进行Transwell实验并分析SCLC的侵袭能力（×200）；E、F：SBC-2（E）和H446^OE^（F）划痕愈合情况及分析（×400）。^****^P<0.0001。

### 2.4 Abemaciclib对SCLC免疫检查点及细胞周期的作用

如图4所示，Abemaciclib明显上调SBC-2（图4A）和H446^OE^（图4B）表面PD-L1的表达并抑制SBC-2（图4C、图4D）和H446^OE^（图4E、图4F）G_1_期-S期细胞周期的进程。

**图4:**
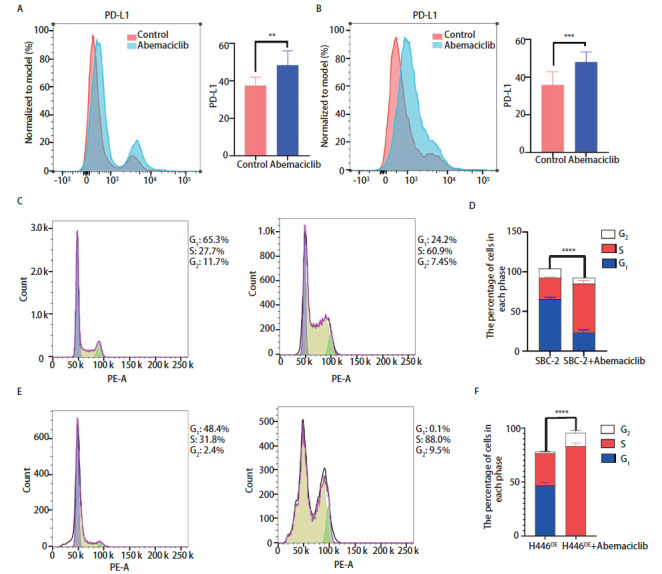
Abemaciclib对SCLC的PD-L1和细胞周期的影响。 A、B：Abemaciclib对SBC-2（A）和H446^OE^（B）表面PD-L1的调节作用及分析；C-F：Abemaciclib对SBC-2（C、D）和H446^OE^（E、F）细胞周期的影响及分析。^**^P<0.01；^***^P<0.001；^****^P<0.0001。

### 2.5 Abemaciclib抑制SCLC细胞系增殖、侵袭和迁移的机制

为了进一步探索Abemaciclib对SCLC的作用机制，我们通过Western blot定量分析了SCLC细胞系中CDK4/6、c-Myc、NEUROD1、YAP1和ASCL1的表达。如图5A和图5B所示，在应用Abemaciclib后，SBC-2和H446中CDK4/6、c-Myc、YAP1、NEUROD1和ASCL1均明显下调。H446^OE^中CDK6、c-Myc、YAP1、NEUROD1和ASCL1的表达均高于H446^NC^，且H446^OE^内的蛋白对Abemaciclib的下调作用更为敏感（图5C、图5D）。

**图5:**
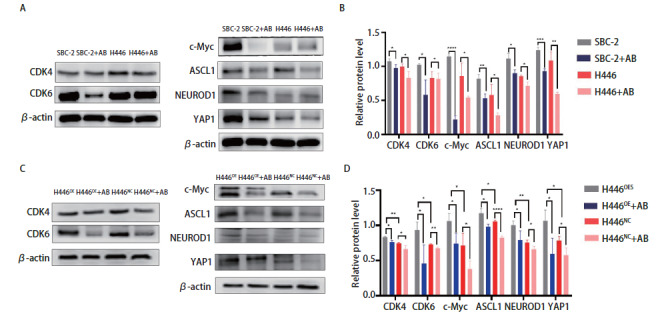
Abemaciclib抑制SCLC细胞增殖、侵袭和迁移的机制。 A、B：Abemaciclib对SBC-2和H446中的CDK4/6、c-Myc、ASCL1、NEUROD1和YAP1的下调作用；C、D：Abemaciclib对H446^OE^和H446^NC^中CDK4/6、c-Myc、 ASCL1、NEUROD1和YAP1的表达调节。^*^P<0.05；^**^P<0.01；^***^P<0.001；^****^P<0.0001。

## 3 讨论

大多数SCLC患者在就诊时就已经进展为广泛期，且预后较差。随着免疫治疗的发展，非小细胞肺癌、结直肠癌和黑色素瘤患者在应用免疫检查点抑制剂时预后均得到改善。但SCLC患者在应用PD-L1抗体Atezolizumab治疗后仅提高了2个月的中位无进展生存期，这可能与SCLC复杂的转录因子及免疫应答低下相关。因此如何抑制SCLC增殖、浸润和转移，改善SCLC免疫微环境从而延长SCLC患者的生存期是目前亟需解决的问题。

c-Myc基因扩增是SCLC最常见的突变之一^[28]^，c-Myc的激活使得原始神经内分泌细胞驱动为不同亚型^[29]^。CDK4/6作为细胞通路的始动因子，通过CDK6/miR-29b-3p/c-Myc轴间接影响c-Myc的表达，促进肿瘤进展。本研究根据STRING蛋白相互作用网络分析结果，通过免疫组化实验证实了CDK4/6及c-Myc在SCLC癌组织中高表达，CDK4/6可以作为Abemaciclib治疗c-Myc高表达SCLC的一个有效靶点。接下来，我们分析了Abemaciclib对高表达c-Myc的SCLC细胞系SBC-2和c-Myc过表达SCLC细胞H446^OE^的增殖、侵袭和迁移的影响。实验结果表明在应用Abemaciclib后，SBC-2和H446^OE^的生物学行为受到抑制，这两种细胞系中CDK4/6的表达显著下调、细胞周期进程阻滞，c-Myc、ASCL1、NEUROD1和YAP1的表达也受到抑制。

ASCL1作为碱性螺旋-环-螺旋家族的成员在多种SCLC细胞系中表达量非常高，其通过协同调控Wnt11的乙酰化促进了神经内分泌因子的分化和细胞增殖^[30]^。和ASCL1一样，NEUROD1在肺神经内分泌因子形成和促进细胞增殖的进程中也起着重要作用^[31]^。Song等^[32]^通过对比53例SCLC患者肿瘤组织发现YAP1不仅与患者的存活率呈负相关，还通过CD74相关信号通路促进肿瘤转移。通过Western blot分析了在应用Abemaciclib后H446^OE^和H446^NC^中ASCL1、NEUROD1和YAP1的表达量，验证了STRING数据库中CDK4/6-c-Myc在SCLC中调节ASCL1、NEUROD1和YAP1的作用。因此，Abemaciclib不仅解决了SCLC转移的问题，也为多种类型癌细胞共存的SCLC提供了一个新的治疗思路。

PD-L1抑制剂作为一种最常见的免疫检查点抑制剂，通过与肿瘤细胞表面的PD-L1结合激活了免疫细胞杀伤肿瘤的作用。肿瘤细胞表面PD-L1高表达的患者在接受PD-L1抗体治疗后能够得到更好的临床获益，如聚二磷酸腺苷核糖聚合酶（poly adenosine diphosphate ribose polymerase, PARP）抑制剂氟唑帕利通过上调PD-L1的表达提高了PD-L1抗体SHR-1316在二线治疗广泛期SCLC的疗效^[33]^。Zhang等的研究^[27]^发现CDK4/6抑制剂通过CDK4上调了PD-L1的表达。本研究结果也表明Abemaciclib可以有效地提高SCLC患者肿瘤细胞表面PD-L1的表达，为Abemaciclib联合PD-L1抗体治疗SCLC提供了理论依据。

综上，CDK4/6作为SCLC治疗的一个有效靶点与c-Myc共同促进SCLC的发生发展。CDK4/6抑制剂Abemaciclib不仅影响了c-Myc高表达SCLC的增殖、侵袭、迁移和细胞周期进程及相关蛋白的表达，也参与了肿瘤免疫检查点的调控。我们的研究初步探索了Abemaciclib抑制c-Myc高表达的SCLC增殖、侵袭和迁移的生物学作用及机制，今后我们将继续探索SCLC相关生物学进展机制，以期为SCLC治疗提供更加精准化和个体化的理论基础。
