# 更正声明

**DOI:** 10.3779/j.issn.1009-3419.2023.103.01

**Published:** 2023-04-20

本刊2023年第26卷第2期刊登的题名为“Abemaciclib抑制c-Myc高表达小细胞肺癌增殖、侵袭和迁移的生物学功能的研究”[郭晶晶, 牟迪, 于文文, 等. Abemaciclib抑制c-Myc高表达小细胞肺癌增殖、侵袭和迁移的生物学功能的研究. 中国肺癌杂志, 2023, 26(2): 105-112.] 一文中：因作者原因导致图片有误，第108页图2G、图2H和图2I中的纵坐标刻度线值“0、1、2、3、4”应为“0、3、6、9、12”，作者希望修正如下图。特此更正。作者对此深表歉意。

Erratum: Study on the Biological Function of Abemecilib in Inhibiting the Proliferation, Invasion and Migration of Small Cell Lung Cancer with High c-Myc Expression

Guo JJ, Mu D, Yu WW, *et al*.

Zhongguo Fei Ai Za Zhi, 2023, 26(2): 105-112.

In the version of this article initially published, error appeared on page 108 for reason of the authors. The ordinate scaleline value “0, 1, 2, 3, 4” in Fig 2G, Fig 2H and Fig 2I should be changed to “0, 3, 6, 9, 12”. The authors expect to make corrections and the correct figures are presented as follows. The authors apologize for the error.

**图 2:**
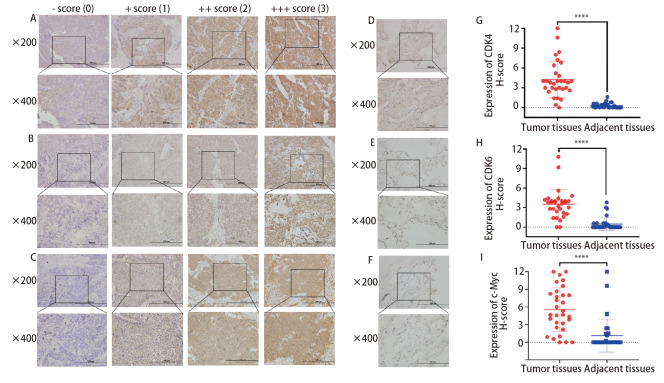
CDK4/6和c-Myc在SCLC组织中的免疫组化染色及评分（×200；×400）。根据H-score评分标准将CDK4（A）、CDK6（B）和c-Myc（C）在SCLC癌组织染色情况进行评分；D-F分别为CDK4、CDK6和c-Myc在癌旁组织中的染色情况。癌组织中CDK4（G）、CDK6（H）和c-Myc（I）的表达均高于癌旁组织。****P<0.0001。

